# Unraveling Mitochondrial Reactive Oxygen Species Involvement in Psoriasis: The Promise of Antioxidant Therapies

**DOI:** 10.3390/antiox13101222

**Published:** 2024-10-11

**Authors:** Hajar Ahmad Jamil, Norwahidah Abdul Karim

**Affiliations:** Department of Biochemistry, Faculty of Medicine, Universiti Kebangsaan Malaysia, Kuala Lumpur 56000, Malaysia; hajarahmadjamil@ukm.edu.my

**Keywords:** psoriasis, mitochondrial dysfunction, mitochondrial reactive oxygen species, oxidative stress, antioxidant, natural products

## Abstract

Psoriasis is a chronic inflammatory skin disorder characterized by immune dysregulation and aberrant keratinocyte proliferation. Despite tremendous advances in understanding its etiology, effective therapies that target its fundamental mechanisms remain necessary. Recent research highlights the role of reactive oxygen species dysregulation and mitochondrial dysfunction in psoriasis pathogenesis. Mitochondrial reactive oxygen species mediate cellular signaling pathways involved in psoriasis, such as proliferation, apoptosis, and inflammation, leading to oxidative stress, exacerbating inflammation and tissue damage if dysregulated. This review explores oxidative stress biomarkers and parameters in psoriasis, including myeloperoxidase, paraoxonase, sirtuins, superoxide dismutase, catalase, malondialdehyde, oxidative stress index, total oxidant status, and total antioxidant status. These markers provide insights into disease mechanisms and potential diagnostic and therapeutic targets. Modulating mitochondrial reactive oxygen species levels and enhancing antioxidant defenses can alleviate inflammation and oxidative damage, improving patient outcomes. Natural antioxidants like quercetin, curcumin, gingerol, resveratrol, and other antioxidants show promise as complementary treatments targeting oxidative stress and mitochondrial dysfunction. This review aims to guide the development of personalized therapeutic methods and diagnostic techniques, emphasizing the importance of comprehensive clinical studies to validate the efficacy and safety of these interventions, paving the way for more effective and holistic psoriasis care.

## 1. Introduction

Psoriasis, a chronic inflammatory skin disorder affecting approximately 2–3% of the global population, manifests as well-demarcated erythematous plaques, scaling, and pruritus [1]. Despite extensive research efforts, the precise etiology of psoriasis remains incompletely understood, posing significant challenges for effective management and treatment. Emerging data in recent years have linked the pathophysiology of psoriasis to dysregulated mitochondrial activity and the production of reactive oxygen species (ROS) as key contributors [2,3,4]. Mitochondria, often referred to as the “powerhouse of the cell”, are essential for apoptosis, calcium homeostasis, and cellular energy metabolism [5]. While low levels of ROS serve as signaling molecules involved in various physiological processes, excessive ROS production can lead to oxidative stress, cellular damage, and inflammation, contributing to the development and progression of chronic inflammatory disorders, including psoriasis [2,4,6,7].

Given that the skin serves as the primary barrier between the body and the external environment, it is particularly susceptible to oxidative stress due to exposure to environmental pollutants, ultraviolet radiation, and microbial pathogens. In psoriasis, dysregulated immune responses and aberrant keratinocyte proliferation drive the formation of characteristic plaques, accompanied by increased oxidative stress and mitochondrial dysfunction within lesional skin [2,4]. Mounting evidence indicates that mitochondrial reactive oxygen species (mtROS) are involved in cellular signaling pathways, immune cell activation, and epidermal hyperplasia, among other aspects of psoriasis [4,7]. Recent studies have also emphasized the possible therapeutic function of antioxidants in regulating levels of mtROS and decreasing oxidative damage in psoriatic lesions [8,9,10]. Antioxidants derived from natural sources, including curcumin, quercetin, gingerol, and resveratrol, have demonstrated potential in reducing inflammation and oxidative stress, indicating their potential as alternative or supplementary therapies for psoriasis [7,11]. Further research is required to completely understand the effectiveness, ideal dosage, and mechanisms of action of these natural chemicals, which can help reduce symptoms and enhance the overall quality of life for affected persons.

This review aims to elucidate the complex role of mtROS in coordinating the pathophysiological cascades that cause psoriasis by combining data from preclinical research and clinical studies. It will explore the molecular mechanisms underlying mtROS generation, their impact on key cellular processes implicated in psoriasis pathophysiology, and the interplay between mitochondrial dysfunction and inflammatory responses in this dermatological condition. By shedding light on potential biomarkers and therapeutic targets, this review seeks to pave the way for precision medicine strategies tailored to individual patients. A deeper understanding of the molecular dynamics regulating mitochondrial function and ROS production in psoriatic skin holds promise for the development of personalized treatment approaches that offer enhanced safety and efficacy for individuals grappling with this debilitating dermatitis. Additionally, this review will examine the potential of antioxidant therapies derived from natural sources to modulate oxidative stress and enhance patient outcomes, emphasizing their role as promising adjuncts in the comprehensive management of psoriasis.

## 2. Mitochondrial Reactive Oxygen Species

Mitochondrial dysfunction and the generation of ROS have garnered increasing attention in the context of psoriasis pathogenesis. This review delves into the background of mitochondrial reactive oxygen species (mtROS) and their intricate relationship with psoriasis, shedding light on their potential as key drivers of disease progression. MtROS are a natural byproduct of cellular respiration that occurs in the mitochondria [5]. When oxidative phosphorylation produces adenosine triphosphate (ATP) as a source of energy, electrons from the electron transport chain escape and react with oxygen, generating ROS such as superoxide anion (O_2_^−^), hydrogen peroxide (H_2_O_2_), and hydroxyl radical (OH) [5,7,12].

While ROS are integral to cell signaling and immune response regulation, their excessive production or inadequate removal can result in oxidative stress [5]. Under physiological conditions, low levels of ROS serve as signaling molecules involved in various cellular processes, including proliferation, differentiation, and immune responses [2,5]. However, excessive or dysregulated mtROS production can disrupt cellular homeostasis, leading to oxidative stress, DNA damage, and mitochondrial dysfunction [2,5,7]. Given mitochondria’s crucial role in cellular metabolism and homeostasis, any imbalance in ROS production and antioxidant capacity due to factors like mitochondrial dysfunction, impaired antioxidant defenses, or environmental stressors can contribute to oxidative damage, implicating diseases such as psoriasis [3].

Psoriasis, characterized by persistent inflammation, abnormal keratinocyte proliferation, and immunological dysregulation, exemplifies an inflammatory condition where mitochondrial dysfunction and mtROS production may play pivotal roles [13]. Firstly, mtROS has been indicated by several studies that it is proven to promote inflammation in psoriasis. The excessive production of ROS can activate pro-inflammatory signaling pathways, such as nuclear factor kappa B (NF-κB) and mitogen-activated protein kinases (MAPKs), prompting the release of pro-inflammatory cytokines and chemokines, thereby perpetuating chronic inflammation in psoriatic skin [7,14,15]. Pro-inflammatory cytokines like interleukin-17 (IL-17A) and tumor necrosis factor-alpha (TNF-α) further stimulate mtROS formation in immune cells and keratinocytes, perpetuating a cycle of inflammation and oxidative stress, worsening disease symptoms [15].

Moreover, mtROS-mediated oxidative stress can promote aberrant keratinocyte proliferation and differentiation, contributing to epidermal hyperplasia and plaque formation in psoriatic skin. As the primary site of psoriatic lesions, the skin is subject to inflammatory stimuli and environmental stressors that impair mitochondrial activity and increase ROS production in keratinocytes [3]. The bidirectional relationship between mtROS and psoriasis involves inflammation and cellular dysfunction exacerbating mitochondrial dysfunction and ROS production. Additionally, ROS and mtROS-induced oxidative damage disrupt normal cellular processes, triggering apoptosis, which, while essential for maintaining tissue homeostasis, can contribute to tissue damage and inflammation, as observed in psoriatic lesions [16].

Oxidative stress, a hallmark feature of psoriasis, results from an imbalance between ROS production and antioxidant defense mechanisms. The generation of mtROS can overwhelm the cell’s antioxidant capacity, hence intensifying oxidative damage in psoriatic skin [17]. This imbalance can occur due to several factors, including increased ROS production from activated immune cells, mitochondrial dysfunction, and decreased antioxidant levels or activity [17,18]. Addressing this imbalance through therapeutic strategies aimed at restoring antioxidant defenses holds promise for managing psoriasis by reducing inflammation and oxidative damage. Overall, the intricate interplay between ROS production, antioxidant defense mechanisms, and mitochondrial dysfunction plays a significant role in psoriasis pathogenesis, emphasizing the potential of antioxidant-based therapeutic approaches for disease management.

## 3. Oxidative Stress Biomarkers Involved in Psoriasis Inflammation

Oxidative stress biomarkers play a crucial role in unraveling the complexities of psoriasis inflammation and its progression. The damaging effects of mtROS on cellular function and viability underscore the significance of mitochondrial dysfunction and oxidative stress in psoriasis pathophysiology. Psoriasis presents a formidable challenge in both research and clinical management due to its heterogeneous nature and variable treatment responses among patients. While protocols such as those developed by the Ministry of Health Malaysia offer comprehensive frameworks for managing psoriasis, they may not effectively address the diverse needs of all patients. Biomarkers serve as invaluable tools in confronting these challenges, providing insights into disease mechanisms, aiding in diagnosis, predicting treatment responses, and identifying personalized therapeutic targets. Existing biomarkers in psoriasis research predominantly focus on inflammation, keratinocyte proliferation, and immune cell activation. Notable examples include cytokines such as TNF-α and IL-17, as well as markers like keratin 16 and Ki67, which have been extensively studied for their associations with disease severity and treatment outcomes [7,14,15]. However, the specificity, sensitivity, and clinical applicability of these biomarkers remain limited, necessitating exploration into new avenues.

**Figure 1:**
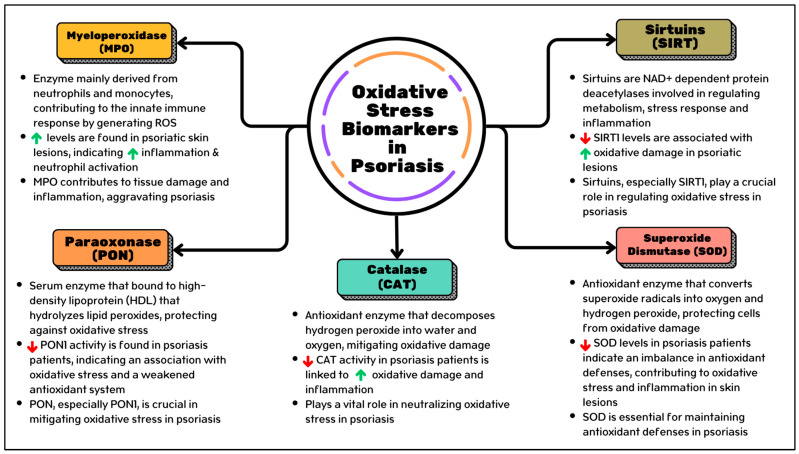
Summary of oxidative stress biomarkers in psoriasis inflammation. This figure illustrates key oxidative stress biomarkers involved in the inflammatory processes of psoriasis. The biomarkers depicted, including myeloperoxidase (MPO), paraoxonase (PON), sirtuins (SIRTs), superoxide dismutase (SOD), and catalase (CAT), are emerging as critical indicators of psoriasis pathogenesis. The red arrows indicate decreasing, while green arrow represent increasing.

In this regard, biomarkers originating from mtROS represent promising novel directions in understanding psoriasis. These biomarkers are poised to become pivotal players in deciphering the intricate interplay between mitochondrial dysfunction, oxidative stress, and psoriasis pathogenesis. Their significance in the pathophysiology of psoriasis will be elucidated through comprehensive analyses spanning preclinical and clinical studies. Noteworthy biomarkers such as myeloperoxidase (MPO), paraoxonase (PON), sirtuins (SIRTs), superoxide dismutase (SOD), and catalase (CAT) are emerging as key indicators of psoriasis pathogenesis and their association with mitochondrial ROS. Through continued investigation and validation, these biomarkers hold the potential to revolutionize the diagnosis, prognosis, and management of psoriasis, paving the way for more targeted and personalized therapeutic approaches. The summary of oxidative stress biomarkers in psoriasis inflammation is displayed in Figure 1. 

### 3.1. Myeloperoxidase (MPO)

Myeloperoxidase (MPO) serves as a pivotal enzyme primarily derived from neutrophils, key players in the immune response, and, to a lesser extent, from monocytes. It plays a crucial role in the innate immune system’s defense against pathogens, particularly bacteria and fungi. Upon activation, neutrophils release MPO, which catalyzes the conversion of hydrogen peroxide (H_2_O_2_) and chloride ions (Cl^−^) into hypochlorous acid (HOCl), a potent oxidizing agent, thereby generating reactive oxygen species (ROS) to combat invading pathogens and support host defense mechanisms [19]. The production of ROS and hypochlorous acid (HOCl) during an inflammatory response via MPO contributes to oxidative stress that damages mitochondrial DNA and impairs mitochondrial function within the skin [20]. Given that MPO serves as a biomarker for the activation of neutrophils and monocytes, the oxidative stress caused by elevated MPO can worsen mitochondrial dysfunction, thereby contributing to the psoriasis etiopathogenesis [20].

In the context of psoriasis, dysregulated immune responses result in the infiltration of activated neutrophils into affected skin lesions. Elevated MPO levels have been observed in psoriasis-related skin lesions, indicating heightened inflammation and neutrophil activation. Excessive or dysregulated MPO activity can lead to tissue damage and contribute to the pathogenesis of various inflammatory conditions, including autoimmune diseases like psoriasis [20]. A study conducted observed that the MPO levels were greater in psoriasis patients’ serum and skin compared to controls [20]. A study on children with psoriasis showed that they had significantly higher MPO levels and activity than healthy controls [21]. A study also observed that psoriatic lesions have elevated MPO levels, which positively correlate with the severity of the illness [22]. This heightened MPO activity can lead to tissue damage and exacerbate the inflammatory processes underlying psoriasis. 

MPO-derived ROS contribute to oxidative damage to lipids, proteins, and DNA, crucial steps in psoriasis pathogenesis that perpetuate inflammatory processes. Tracking MPO activity and levels provides insights into the extent of oxidative stress and inflammation in psoriasis, serving as potential biomarkers for disease severity and progression. Targeting MPO expression or activity presents a potential therapeutic approach to mitigate inflammation and oxidative damage in psoriasis and other inflammatory conditions. In a study using a mouse model of plaque psoriasis induced by Aldara (imiquimod) cream, researchers investigated the impact of MPO inhibition on psoriatic skin lesions [23]. Researchers created an innovative method for evaluating the severity of psoriasis in mice. They discovered that administering MPO inhibitors, both through the bloodstream and directly to the affected area, resulted in a decrease in psoriasis severity [23]. This finding indicates that targeting oxidative damage might be a promising therapeutic strategy for treating psoriasis.

Research shows that MPO gene mutations affect neutrophil activity, inflammation control, and skin inflammation resolution, highlighting the genetic basis of generalized pustular psoriasis (GPP) and related disorders. Variants that cause MPO deficit and decrease MPO protein function have been found in individuals with acute generalized exanthematous pustulosis (AGEP), acral pustular psoriasis (APP), and GPP. A study initially discovered that patients with GPP or APP have a specific genetic mutation called c.2031-2A>C homozygous mutation [24]. This mutation occurs due to a change from A to C in the 3′ end of intron 11 in the MPO gene. As a result of this mutation, a hidden 3′ splice site located 109 base pairs before the usual 3′ splice site becomes activated. This activation causes the insertion of a 119-base-pair fragment and a shift in the reading frame, which ultimately leads to the premature truncation of the protein. Next, a recent study discovered a specific genetic mutation (c.1769G>T, p. Arg590Leu) in the MPO gene of a Japanese patient with GPP. This mutation caused a complete absence of myeloperoxidase activity and decreased levels of MPO protein in the skin [25]. These findings emphasize the important contribution of MPO gene mutations to the development of GPP. Haskamp et al. confirmed the substantial contribution of MPO gene abnormalities to the progression of GPP by discovering many loss-of-function mutations in the MPO gene among afflicted people. The mutations, which included homozygous and compound heterozygous variations, were shown to be linked to decreased MPO activity [26]. This suggests that there may be a possible mechanism that contributes to the development of GPP.

In conclusion, MPO plays a significant role in oxidative stress, inflammation, and tissue destruction in psoriasis-affected skin lesions, contributing significantly to the disease’s pathogenesis. Enhanced MPO activity in psoriatic lesions leads to ROS generation, further exacerbating oxidative stress and perpetuating inflammatory cascades. The identification of MPO gene mutations in patients with GPP further highlights the significance of MPO in psoriasis pathogenesis, offering potential avenues for targeted therapeutic interventions to mitigate inflammation and oxidative damage.

### 3.2. Paraoxonase (PON)

Paraoxonase (PON), a serum enzyme bound to high-density lipoprotein (HDL), plays a crucial role in hydrolyzing organic phosphates and lipid peroxides, thereby protecting against LDL oxidation and potentially reducing disease risks [27,28]. The paraoxonase family comprises PON1, PON2, and PON3, all of which are connected with mitochondria-associated membranes (MAMs) [29]. MAMs have a crucial function in multiple cellular processes, such as regulating mitochondrial metabolism by safeguarding mitochondrial membranes against oxidative stress. This ensures the correct functioning of mitochondria and helps to decrease the formation of mtROS [29]. Among the variants of PON, psoriasis patients were found to have lower PON1 activity in their blood compared to healthy individuals, indicating that psoriasis is linked to oxidative stress and a weakened antioxidant system. This may contribute to the development and advancement of psoriasis and its associated complications [30].

PON1 is a glycoprotein with a molecular weight of 43–45 kDa and it is situated on the q21-q22 region of the long arm of chromosome 7 in humans [31]. The two primary variations of PON1 are L55M and Q192R, in which Leucine (L) at position 55 is substituted with Methionine (M), and Glutamine (Q) at position 192 is replaced with Arginine (R), respectively [30]. The paraoxonase activities of the 55 M and 192 R isozymes have been documented to exhibit lower levels compared to the 55 L and 192 Q enzymes [32]. There is an inverse relationship between serum PON1 activity and serum HDL susceptibility to oxidation in individuals [30].

The PON1 gene displays genetic variations that impact the activity, stability, and selectivity of the enzyme. A study investigates the association between PON1 polymorphisms (rs662 and rs854560) and psoriasis susceptibility in a western Mexico population involving 104 psoriasis patients and 124 controls [27]. The results demonstrate elevated lipid profile levels in patients, accompanied by reduced paraoxonase and arylesterase activity. According to the study conducted by Hernández-Collazo et al. (2020), the G allele of rs662 is correlated with a higher chance of developing psoriasis, whereas the T allele of rs854560 is connected with a reduced susceptibility to the condition. In addition, a research study examines the correlation between the PON1 55 M allele and psoriasis in a case-control study involving 100 individuals with psoriasis and 100 healthy individuals [33]. The presence of the PON1 55 M allele was determined to be associated with psoriasis using polymerase chain reaction–restriction fragment length polymorphism. Patients with the PON1 M allele showed increased levels of MDA, as well as decreased arylesterase (ARE) activity. This indicates a connection between oxidative stress, impaired antioxidant defense, and the development of psoriasis [33].

Reduced PON1 activity in individuals with psoriasis implies its role in disease pathology, contributing to oxidative stress and inflammation by disrupting the balance between ROS production and antioxidant defenses. Studies have demonstrated correlations between PON levels, disease severity, and chronic inflammation markers in psoriasis patients. In correlation with psoriasis severity, Ferretti et al. observed diminished activity of PON1 and arylesterase and they also noted a negative relationship between lipoprotein levels and PON1 activity, indicating that individuals with higher lipoprotein levels may experience increased susceptibility to oxidative damage [34]. Similarly, lower PON1 activity correlated with higher lipoprotein levels, exacerbating oxidative stress susceptibility in psoriasis patients [35]. This decrease, which was positively correlated with the severity of the condition, might be the consequence of ROS-induced antioxidant inactivation [35].

Furthermore, its robust antioxidant capabilities enable it to neutralize ROS and hydrolyze lipid peroxides, thereby mitigating oxidative stress. Additionally, PON acts as an antioxidant enzyme by dismantling and neutralizing hazardous lipid peroxides produced during oxidative stress, thus preventing tissue and cellular damage [27]. Notably, studies have investigated the antioxidant role of PON-1 and vitamin E in psoriasis, revealing reduced PON1 levels in psoriasis patients compared to controls, suggesting a potential association between decreased PON1 activity and psoriasis onset [36]. Beyond its antioxidant properties, PON exerts anti-inflammatory effects, further bolstering its significance in autoimmune diseases, including psoriasis. Studies have shown decreased PON activity alongside elevated oxidative stress and inflammation in psoriasis patients, indicating the involvement of PON in the disease pathogenesis [21]. Fifty-two psoriatic patients and 48 sex-age-matched healthy controls were included in a study to assess PON1 activity in psoriatic children [21]. Notably, treatment with TNF-α inhibitors improved clinical outcomes in psoriasis patients by enhancing PON1 activity, underscoring the link between PON1 activity and inflammation [37]. Etanercept treatment improved clinical outcomes, decreased lipid peroxidation and inflammation, and raised antioxidant capacity [37].

Overall, PON plays a critical role in psoriasis pathogenesis by mitigating oxidative stress and inflammation in affected skin lesions. Alterations in PON activity contribute to the dysregulation of redox homeostasis, exacerbating oxidative stress, inflammation, and tissue damage in psoriasis. Understanding the complex relationship among PON, oxidative stress, and inflammation provides insights into possible treatment approaches for psoriasis management and enhancing patient results. 

### 3.3. Sirtuins (SIRTs)

Sirtuins are a family of highly conserved NAD+-dependent protein deacetylases that regulate physiological functions like metabolism, stress response, inflammation, and aging. Encoded by the SIRT1-7 genes in mammals, these enzymes exhibit tissue-specific expression patterns and diverse subcellular localization [38]. SIRT1 and SIRT2 are located in the cytoplasm and nucleus of the cell, whereas SIRT3–SIRT5 are found in the mitochondria, and SIRT6–SIRT7 are nuclear proteins [38]. In terms of function, SIRT1 controls signaling pathways linked to inflammation and suppresses oxidative stress, mitochondrial DNA mutation, mtROS, and mitochondrial damage. SIRT1, SIRT2, and SIRT6 regulate metabolism and lifespan by altering the NF-kB and fatty acid ß-oxidation pathways, whereas SIRT3 enhances fatty acid ß-oxidation and activates critical electron transport chain and urea cycle enzymes [39]. While SIRT7 is involved in DNA damage repair, inflammatory cytokine release, and cell survival, SIRT4 and SIRT5 activate enzymes that control cellular metabolism, such as the pyruvate dehydrogenase (PdH) complex and the glutamate dehydrogenase (GdH) complex in particular [40,41].

Research used both in vitro and in vivo models, such as imiquimod-induced psoriasis in mice and TNF-stimulated HaCaT cells, to examine the expression levels and patterns of SIRTs in psoriasis. Dysregulation of sirtuin expression, observed in psoriasis with reductions in some (SIRT1-5) and increases in others (SIRT6, SIRT7), was reported to contribute to oxidative stress and inflammatory processes associated with the condition [42]. Additionally, the study revealed that SIRTs are mostly located in the epithelial layer, indicating that keratinocytes, acting as antigen-presenting cells instead of lymphocytes, might be the main immune response initiator [42]. Emerging evidence suggests that sirtuins, particularly SIRT1, may play a role in the pathogenesis of psoriasis, a chronic inflammatory skin disorder characterized by abnormal immune responses and skin cell proliferation. Through its ability to modulate the activity of transcription factors like NF-κB and AP-1, which are critical in controlling inflammatory responses, SIRT1 demonstrates anti-inflammatory capabilities and has been linked to guarding against oxidative stress-induced damage in psoriasis. By inhibiting pathways including MAPK, NF-κB, and STAT3, which are all linked to oxidative stress-induced inflammation, SIRT1 activation might potentially reduce inflammation in psoriatic skin [43]. 

According to research, skin biopsy specimens from psoriasis patients have altered SIRT expression, with significantly lower SIRT1 expression than control skin samples [44]. It is reported that nearly every nucleus in the epithelial layer of skin samples from healthy controls had a positive SIRT1 stain, whereas psoriatic samples were rare and with very weak staining showed this effect [44]. Other than that, the SIRT1 activator SRT2104 exhibits promise in mitigating psoriasis symptoms by reducing the expression of genes linked to inflammatory responses and skin cell differentiation, which are IL-17 and TNF-α [18,45,46]. These results point to a possible therapeutic route for controlling psoriasis via SIRT-mediated pathways, indicating the need for more research into SIRT activators as cutting-edge psoriasis treatments. This shows that by focusing on oxidative stress and inflammatory pathways, sirtuins may provide therapeutic options for controlling psoriasis.

Sirtuins may assist in preserving redox balance and shielding cells from oxidative damage in psoriasis, a condition where there is an imbalance between the generation of ROS and antioxidant defenses [47]. They achieve this by regulating the expression and activity of antioxidant enzymes and pro-oxidants [48]. Sirtuins protect cells from oxidative damage by balancing pro-oxidant radicals with antioxidant enzymes such glutathione peroxidases (GSH-pxs), CAT, and SOD [47,48]. In particular, SIRT1 is critical for reducing damage caused by oxidative stress because it suppresses the generation of ROS in the mitochondria, which is a defense mechanism for cells against oxidative assaults that worsen psoriatic inflammation. Psoriatic lesions have been shown to exhibit dysregulation of sirtuin expression, notably decreased SIRT1 levels, indicating a possible connection between sirtuins and oxidative stress in the pathophysiology of the illness.

In summary, sirtuins, particularly SIRT1, play a significant role in modulating oxidative stress in psoriasis by regulating antioxidant defenses, inhibiting inflammatory pathways, and maintaining redox balance. Understanding the intricate interplay between sirtuins, oxidative stress, and psoriasis not only sheds light on disease mechanisms but also offers promising avenues for therapeutic intervention. Psoriatic inflammation and related oxidative stress may be effectively managed by utilizing the therapeutic potential of sirtuin regulation. With their anti-inflammatory and antioxidant qualities, studies on sirtuin activators may be able to reduce the symptoms of psoriasis and enhance the quality of life for affected individuals.

### 3.4. Superoxide Dismutase (SOD)

Superoxide dismutase (SOD) is a vital enzyme that aids in antioxidant defense inside cells. Its principal function is to catalyze the dismutation of superoxide radicals (O_2_^−^) to oxygen (O_2_) and hydrogen peroxide (H_2_O_2_) [49]. This enzymatic process is essential for defending cells against the damaging effects of ROS and oxidative stress. SOD exists in three main forms: cytosolic (SOD1), mitochondrial (SOD2), and extracellular (SOD3), each encoded by distinct genes [49]. SOD2, specifically found in the mitochondria, has a vital function in mitigating mtROS by converting superoxide radicals [50]. The dismutation of superoxide radicals by SOD is an important step in the antioxidant defense mechanism of cells. SOD protects cellular components such as proteins, lipids, and DNA from oxidative damage by converting superoxide radicals into less damaging molecules like oxygen and hydrogen peroxide. This mechanism is critical to maintaining cellular redox equilibrium and general health. Deficiencies or dysregulation of SOD activity, particularly SOD2, leads to increased mtROS and oxidative stress, which have been associated with various diseases and conditions, including psoriasis. Therefore, SOD plays a significant role in protecting cells from oxidative damage and maintaining their normal function and integrity. 

In psoriasis, where there is an imbalance between ROS production and antioxidant capacity, SOD helps to scavenge superoxide radicals and reduce oxidative stress. The observed alterations in SOD levels among individuals with psoriasis underscore the complex nature of oxidative stress in this skin condition. Several studies indicate a decrease in SOD activity, suggesting a compromised antioxidant defense system in affected individuals. This decrease in SOD levels may lead to an imbalance between ROS generation and antioxidant capacity, worsening oxidative stress and inflammation in the skin and so impacting the severity and course of psoriasis. In 2020, Khan et al. noted a correlation between SOD levels and psoriasis, finding significantly lower SOD levels in psoriatic patients compared to healthy controls [51]. Similarly, Kaur et al. (2016) also investigated SOD levels in both psoriasis patients and controls, reporting markedly decreased SOD levels (168.46 ± 51.89 U/mL) in psoriasis patients compared to the control group (237 ± 39.30 U/mL) [52]. Plasma levels of SOD were evaluated in 100 psoriasis patients and 100 controls revealed a significant decrease in SOD activity (*p* < 0.05) among psoriasis patients, suggesting a state of oxidative stress in the disease [35]. These findings imply a potential link between diminished SOD activity and psoriasis development. 

However, research examining the levels of SOD in the saliva and blood of plaque psoriasis patients to healthy controls revealed considerably greater SOD concentrations in both unstimulated and stimulated saliva of psoriasis patients, suggesting redox imbalances and oxidative stress in the condition [53]. Research also found that the activity of SOD1 was notably elevated in the plasma of individuals with psoriasis [54]. Besides that, the levels of SOD1 and SOD2 genes were measured using real-time PCR, as described by Esmaeili et al. (2018). No notable disparities were detected in the manifestation of SOD1 or SOD2 genes among patients and controls, as well as across various clinical subtypes of psoriasis [55]. These investigations have shown conflicting results, with no significant decreases in SOD levels detected in psoriasis patients vs. healthy controls. 

These disparities might be attributable to variations in research demographics, illness severity, sample collecting procedures, or analytical methodologies. Fluctuations in SOD gene expression or enzyme activity may contribute to the disruption of cellular redox equilibrium, intensifying oxidative stress and promoting the advancement of illness. Additionally, genetic variances, environmental triggers, and comorbidities may all contribute to the diversity in SOD levels among psoriasis patients. Understanding the dynamics of SOD levels in psoriasis is crucial for unraveling the intricate mechanisms underlying oxidative stress and inflammation in this condition. Further research is needed to elucidate the precise role of SOD in psoriasis pathogenesis and its potential as a therapeutic target for managing the disease. Clarifying these relationships may open the way for the development of specific therapies aiming at restoring SOD activity and strengthening antioxidant defenses in order to ease symptoms and enhance outcomes in psoriasis patients.

In summary, considering the crucial function of SOD in counteracting ROS, implementing measures to restore its activity and alleviate oxidative stress may provide significant advantages in the treatment of psoriasis and the reduction of skin inflammation. Interventions that aim to increase the activity of SOD, which helps protect against damage caused by ROS, have the potential to reduce symptoms and enhance the overall quality of life for people with psoriasis. These findings highlight the significance of conducting more research to create personalized therapy methods for managing psoriasis by focusing on SOD.

### 3.5. Catalase (CAT)

Catalase (CAT) is an essential antioxidant enzyme found in nearly all oxygen-exposed living organisms, serving to protect cells from the damaging effects of ROS by breaking down hydrogen peroxide (H_2_O_2_) into water (H_2_O) and oxygen (O_2_) [56,57]. With a heme group in its active site, catalase facilitates the conversion of hydrogen peroxide, preventing its accumulation and subsequent oxidative damage to cellular components such as proteins, lipids, and DNA [57]. Catalase protects the mitochondria and the cell by regulating the amounts of H_2_O_2_, thereby preventing oxidative stress and promoting optimal mitochondrial function. Deficiencies in catalase activity or expression have been associated with increased susceptibility to oxidative stress, mitochondrial DNA damage and various diseases, highlighting its crucial role in cellular defense mechanisms [58]. 

Psoriasis is characterized by increased oxidative stress, which refers to an imbalance between the generation of damaging ROS and the body’s antioxidant defenses. The uneven distribution causes oxidative harm to cellular structures, intensifying inflammation and playing a role in the formation of psoriatic lesions. Catalase, as a key component of the body’s antioxidant defense system, plays a vital role in mitigating the impact of oxidative stress by catalyzing the breakdown of hydrogen peroxide, thereby preventing the accumulation of reactive oxygen species and protecting skin cells from oxidative damage [57]. Maintaining optimal catalase activity is essential in psoriasis to mitigate the harmful effects of oxidative stress, thereby preserving skin integrity and reducing disease progression. A study assessed the plasma levels of CAT in 100 psoriasis patients and 100 healthy controls resulted in a significant reduction in CAT activity (*p* < 0.05) among individuals with psoriasis, suggesting the presence of oxidative stress in the condition [35].

A study aimed to assess the redox imbalance and protein changes in lymphocytes of individuals diagnosed with psoriasis vulgaris (PsV) or psoriatic arthritis (PsA) [59]. The findings indicated a more pronounced transition towards circumstances that promote oxidation in lymphocytes of individuals with psoriatic arthritis (PsA). This was characterized by elevated levels of reactive oxygen species and reduced catalase activity in comparison to patients with PsV and healthy individuals [59]. Subsequently, research was conducted to evaluate the level of oxidative stress in individuals with psoriasis by quantifying the presence of malondialdehyde (MDA) in their blood serum as an oxidant, as well as assessing the activity of erythrocyte CAT as an antioxidant [60]. The study found that psoriasis patients had significantly lower erythrocyte catalase activity (*p* < 0.001) compared to the control group [60]. These findings imply that oxidative stress plays a role in the development of psoriasis.

The link between CAT gene expression and psoriasis was also investigated. The catalase gene in humans was discovered and described by Quan et al. in 1986 [61]. It is located on chromosome 11, specifically on band p13, and consists of 13 exons and 12 introns. The presence of regulatory components inside it allows for the modulation of its expression in response to different cellular signals, such as oxidative stress. Comprehending these regulatory processes is essential for interpreting the function of catalase in disorders like psoriasis. A study observed the quantification of CAT gene expression was conducted using real-time PCR, following the methodology outlined by Esmaeili et al. (2018). The study by Esmaeili et al. (2018) discovered a significant reduction in the expression of the catalase gene (*p* = 0.02) in psoriasis patients, suggesting that catalase may play a part in the redox imbalance linked to psoriasis. Other than that, a study used data mining and bioinformatic scripting to examine the genomic impacts of psoriasis, specifically gene expression in afflicted tissues [62]. Research suggests that psoriasis may lead to reduced CAT activity, which contributes to oxidative stress and may exacerbate the disease’s etiology [62].

Ultimately, understanding the complex correlation between catalase and psoriasis is crucial for developing efficient therapy strategies that specifically address oxidative stress and enhance antioxidant defenses in patients. Enhancing catalase activity and alleviating oxidative stress may have potential in the management of psoriasis and the reduction of skin inflammation. The relevance of catalase in antioxidant defense systems is evident in its function in protecting skin cells from oxidative damage, regulating inflammation, and perhaps influencing the severity and development of psoriatic lesions. Therefore, the use of CAT activity or expression as a therapeutic approach might provide new possibilities for the treatment of psoriasis and the management of oxidative stress-related pathways involved in the illness.

## 4. Oxidative Stress Parameters Involved in Psoriasis Inflammation

**Figure 2:**
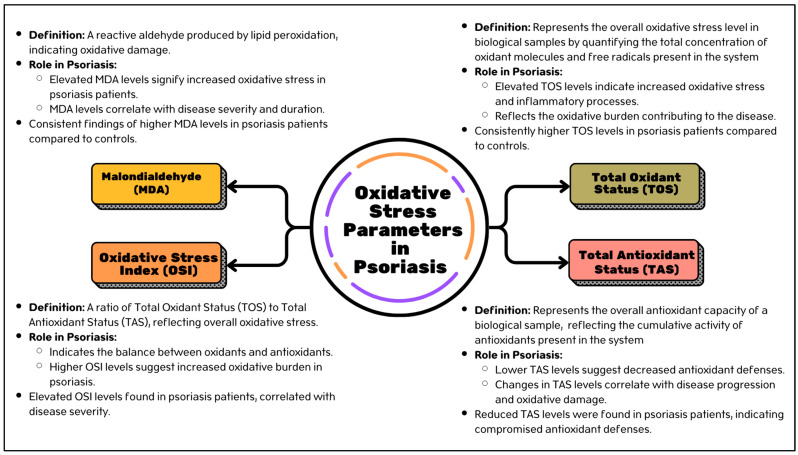
Overview of oxidative stress parameters in psoriasis inflammation. This figure provides a summary of key oxidative stress parameters associated with psoriasis. It depicts the roles of malondialdehyde (MDA), total antioxidant status (TAS), oxidative stress index (OSI), and total oxidant status (TOS) in evaluating oxidative stress and antioxidant defense mechanisms in psoriasis patients.

Amidst the complexity of psoriasis pathophysiology, parameters become vital instruments for unraveling the fundamental molecular processes propelling the advancement of the illness. Understanding the parameters linked to psoriasis can provide significant understanding of its fundamental causes and facilitate the development of targeted therapy approaches. In this regard, parameters such malondialdehyde (MDA), total antioxidant status (TAS), oxidative stress index (OSI), and total oxidant status (TOS) become essential instruments for evaluating antioxidant defense mechanisms and oxidative stress in psoriasis patients. The roles of MDA, OSI, TOS, and TAS as parameters for oxidative stress dynamics, disease severity, and medication response are examined. The complex interactions that exist between these markers and the pathophysiology of psoriasis by thorough analysis will pave the way to more specialized and individualized treatment modalities. The summary of oxidative stress parameters involved in psoriasis inflammation is displayed in Figure 2.

### 4.1. Malondialdehyde (MDA) 

Malondialdehyde (MDA) is a reactive aldehyde compound generated as a byproduct of lipid peroxidation, a process initiated when free radicals attack unsaturated fatty acids within cell membranes [63]. Widely recognized as a marker of oxidative stress, MDA levels serve as indicators of oxidative damage in biological systems. Elevated MDA levels signify increased lipid peroxidation and oxidative stress, processes implicated in the pathophysiology of various disorders, including inflammatory skin conditions like psoriasis [64]. In studies involving psoriasis patients, elevated MDA levels have been consistently observed, indicating heightened oxidative stress in this population [47,65]. In a study conducted by Singal et al., 150 psoriasis patients and 150 healthy controls had their serum lipids, lipoproteins, and antioxidant–oxidant status assessed. The findings revealed that the psoriasis patients had significantly higher levels of lipid peroxidation products, such as MDA [66]. In addition, 23 psoriasis patients and 23 healthy people had their levels of MDA analyzed and the findings indicated that the MDA levels in the psoriasis patients were considerably greater than those in the control group [65].

Furthermore, MDA levels have been linked to disease severity and duration in psoriasis patients, suggesting a potential role in disease progression. Fifty psoriasis patients participated in research where their MDA levels were measured [67]. The findings showed that psoriatic patients had much higher MDA levels than controls, and there were positive associations found between MDA levels and the length and severity of the illness [67]. Measurement of MDA levels in biological samples such as blood, urine, and tissues provides valuable insights into oxidative stress status and disease monitoring. A study compared the blood and saliva of patients with plaque psoriasis to healthy controls in order to assess redox balance parameters and indicators of oxidative stress in both stimulated and nonstimulated saliva [53]. MDA concentrations in psoriatic patients’ samples were considerably higher than in controls, suggesting that patients with plaque psoriasis have greater levels of oxidative stress [53]. According to these results, MDA concentration in saliva may be used as a biomarker for plaque psoriasis diagnosis. According to Şikar Aktürk et al.’s study, tissue levels of MDA in lesional skin were significantly higher than in non-lesional skin, indicating a role for increased oxidative stress in the etiology of psoriasis [65]. 

Overall, elevated MDA levels indicate heightened oxidative damage and the need for antioxidant intervention. Monitoring MDA levels offers insights into the degree of oxidative stress and its impact on skin diseases like psoriasis. Beyond serving as oxidative stress markers, MDA actively participates in cellular dysfunction and disease pathogenesis by forming adducts with proteins and DNA, thereby contributing to tissue damage, inflammation, and disruption of vital cellular processes. Understanding the role of MDA in psoriasis pathophysiology provides avenues for targeted therapeutic interventions aimed at mitigating oxidative stress and improving patient outcomes.

### 4.2. Oxidative Stress Index (OSI)

The oxidative stress index (OSI) is a ratio that compares the level of oxidative stress, which is represented by total oxidant status (TOS), to the total antioxidant capacity, which is represented by total antioxidant status (TAS) [68]. Oxidative stress plays a significant role in promoting inflammation in psoriasis. The presence of ROS can activate inflammatory pathways, trigger immune responses, and contribute to the development of psoriatic lesions. Therefore, monitoring OSI levels can help assess the degree of oxidative stress-induced inflammation in psoriasis patients. Measurement of the OSI provides valuable information about the overall redox status and oxidative burden experienced by cells and tissues. In conditions associated with increased oxidative stress, such as psoriasis, the OSI may be elevated, reflecting an imbalance where the production of oxidants surpasses the body’s ability to neutralize them, contributing to the pathogenesis and progression of the disease [53]. On the other hand, a lower OSI score denotes a more advantageous equilibrium in which antioxidant defenses are more effective in counteracting oxidative stress [53]. The OSI offers a quantitative measure of the equilibrium between antioxidant defenses and oxidative stress. The OSI value is calculated according to the following formula: OSI (arbitrary unit) = TOS (μmol H_2_O_2_ Eq\L)\TAS (μmol Trolox Eq\L) × 100 [68].

Studies have shown that psoriasis patients often exhibit higher OSI values compared to healthy individuals. A study evaluated redox balance parameters and oxidative stress biomarkers in plaque psoriasis patients compared to healthy controls [53]. The OSI values in unstimulated and stimulated saliva, as well as plasma of psoriatic patients, were significantly higher (*p* ≤ 0.001), suggesting potential diagnostic biomarkers for plaque psoriasis [53]. The OSI was then used in a study to measure oxidative stress in psoriatic patients. The results showed that psoriasis patients had significantly higher levels of the OSI (*p* < 0.001) than controls, and that these levels positively correlated with the severity and duration of the disease. This suggests that psoriasis may be related to increased reactive oxygen species and decreased antioxidant activity [69]. In addition, a study looked at OSI and inflammatory markers in people with psoriasis vulgaris. The findings showed that psoriasis patients had significantly higher baseline levels of OSI than controls and that OSI and sialic acids significantly correlated with the severity of the disease [70]. Additionally, after 12 weeks of therapy, a significant decline in the OSI and inflammatory markers was seen, indicating a possible link between oxidative stress, inflammation, and the pathogenesis of psoriasis [70].

In individuals with psoriasis, monitoring OSI levels can assist in assessing the severity, course, and responsiveness to therapy of the condition. Treatment options may change if the link between OSI and psoriasis is understood. Psoriasis patients may have less inflammation and better results with therapies targeted at lowering oxidative stress and reestablishing antioxidant balance. To control OSI levels and lessen oxidative stress in psoriasis, lifestyle changes, dietary changes, and antioxidant supplements may be taken into consideration. In summary, the OSI is a helpful biomarker for assessing antioxidant state and oxidative stress in biological systems, providing information on the pathophysiology of oxidative stress-related diseases and potential targets for therapeutic intervention.

#### Total Oxidant Status (TOS) and Total Antioxidant Status (TAS)

Total oxidant status (TOS) and total antioxidant status (TAS) stand as pivotal parameters in evaluating the redox equilibrium within biological systems, shedding light on the interplay between antioxidant defenses and oxidative stress. TOS serves as a comprehensive measure that reflects the overall oxidative stress level in biological samples by quantifying the total concentration of oxidant molecules and free radicals present in the system, while TAS measures the overall antioxidant capacity of a biological sample, reflecting the cumulative activity of antioxidants present in the system [70]. In the context of psoriasis, disturbances in the equilibrium between TOS and TAS have been implicated in disease pathogenesis and progression. 

TOS quantifies the oxidative burden within biological systems, reflecting the levels of oxidant molecules, including ROS and reactive nitrogen species (RNS). The heightened generation of ROS and oxidative stress in psoriatic lesions, stemming from immunological dysregulation and inflammatory processes, is mirrored in elevated TOS levels [53]. Studies consistently demonstrate higher TOS levels in psoriasis patients, indicative of increased oxidative stress and the accumulation of oxidant molecules capable of causing cellular damage [4,53]. In patients with plaque psoriasis, significantly higher values of TOS and OSI were observed in nasal washings (NWS), sweat washings (SWS), and plasma compared to the control group, along with a faster rate of ROS production in NWS and SWS [53]. Psoriasis patients have higher levels of TOS due to several factors, including abnormal immune cell activation, increased synthesis of inflammatory cytokines, and compromised antioxidant defenses.

Conversely, TAS represents the cumulative capacity of endogenous and exogenous antioxidants to neutralize oxidant molecules and maintain redox homeostasis. Antioxidants scavenge free radicals, inhibit lipid peroxidation, and repair oxidative damage, thereby mitigating the detrimental effects of oxidative stress [2,71]. Psoriasis is characterized by alterations in TAS levels, reflecting changes in antioxidant capacity and redox balance. Decreased TAS levels may indicate compromised antioxidant defenses, rendering cells more susceptible to oxidative damage and exacerbating inflammation and tissue injury in psoriatic lesions [2,71]. The study evaluated TAS in individuals with psoriasis and showed that TAS levels were reduced but were not statistically significant before or after methotrexate (MTX) medication [72]. TAS was measured in 20 healthy controls and 55 psoriasis patients based on severity, and the results showed that TAS levels were lower in all psoriasis groups than in the control group [71]. These results emphasize the involvement of antioxidant defense systems in the pathophysiology of the disease and point to a possible correlation between decreased TAS and the severity of psoriasis. 

The delicate balance between antioxidant defenses and oxidative stress is reflected in the link between TOS and TAS dynamics in psoriasis. Imbalances favoring higher TOS and/or lower TAS exacerbate the severity of the illness by prolonging oxidative damage and inflammation in psoriatic lesions. Understanding the interplay between TOS and TAS dynamics provides insights into the underlying mechanisms driving psoriasis pathogenesis. In a study on 40 psoriatic patients, Rajappa et al. observed elevated levels of TOS, highly sensitive C-reactive protein (hs-CRP), total sialic acids [16], and protein-bound sialic acid (PBSA) at baseline, along with decreased of TAS, correlating positively with PASI scores [70]. Following a 12-week treatment with MTX, reductions in TOS, hs-CRP, TSA, and PBSA were noted alongside an increase in TAS levels, indicating potential therapeutic efficacy in modulating oxidative stress parameters in psoriasis [70]. In contrast, a study observed that TOS levels between the patient and control groups demonstrated a significant disparity, while TAS levels did not significantly differ between the two groups. This observation was made in a study involving fifty psoriasis patients who were not receiving systemic medication and forty-five healthy volunteers [73]. Other than that, there is a study that also observed no significant increase or statistically significant alteration in TAS and TOS levels over the 8 weeks of the study [74]. Kılıc et al. (2013) mentioned in their study that it is probably due to a lower dose of MTX or the durations might be too short to cause any changes. Some studies have reported discrepancies in TOS and TAS levels between patient and control groups, underscoring the need for further research to elucidate their precise roles in psoriasis pathophysiology and treatment response [70,73,74]. 

To sum up, the balance between TOS and TAS is crucial for maintaining redox homeostasis in the body. While elevated TOS levels reflect increased oxidative stress and ROS production, decreased TAS levels indicate decreased antioxidant capacity and impaired antioxidant defenses. This imbalance tips the scales in favor of oxidative stress, leading to mitochondrial ROS generation, oxidative damage, and inflammation, perpetuating the disease process in psoriasis. Therefore, TOS and TAS represent valuable biomarkers in assessing oxidative stress status and guiding therapeutic interventions in psoriasis management.

## 5. Antioxidants from Natural Products as Alternative Treatment 

Understanding the intricate molecular mechanisms underlying mtROS-mediated inflammation and cellular dysfunction in psoriasis offers valuable insights into identifying novel therapeutic targets and tailoring individualized therapy plans for affected individuals. Modulating mtROS levels and enhancing antioxidant defenses holds promise as a potential therapeutic strategy in managing psoriasis, with the potential to alleviate inflammation and oxidative damage in psoriatic lesions, thereby improving patient outcomes and quality of life [75,76]. Various strategies may be used to do this, such as increasing antioxidant intake through food or medicine, modifying the activity of antioxidant enzymes, and focusing on the processes that produce reactive oxygen species. These therapies have the potential to lessen oxidative stress and restore redox equilibrium and may help alleviate symptoms and improve outcomes in individuals with psoriasis.

In recent years, there has been growing interest in the potential of antioxidants derived from natural products as alternative treatments for psoriasis [11]. These antioxidants present appealing options for adjunctive therapy in psoriatic lesions due to their ability to target inflammation and oxidative stress. Compounds such as quercetin, curcumin, gingerol, resveratrol, epigallocatechin-3-gallate (EGCG), rutin, and genistein have demonstrated promising effects in managing psoriasis symptoms. Integrating these naturally occurring antioxidants into the therapeutic armamentarium for psoriasis holds the potential for symptom alleviation and overall health enhancement in affected individuals. However, further research and clinical investigation are imperative to elucidate the full potential of natural antioxidants in reducing oxidative stress and psoriatic inflammation. Well-designed clinical studies are warranted to clarify their efficacy, optimal dosage, and long-term safety in the treatment of psoriasis. Additionally, exploring the mechanisms of action underlying the therapeutic effects of these antioxidants will provide valuable insights into their clinical utility and pave the way for the development of more effective and personalized treatment strategies for psoriasis.

### 5.1. Curcumin

Curcumin, a bioactive compound found in turmeric (*Curcuma longa*), has garnered significant attention for its potent antioxidant properties and numerous health benefits. Its ability to scavenge free radicals, modulate oxidative stress, and protect cells from damage makes it particularly relevant in the context of psoriasis pathogenesis. Studies have shown that combination therapy of curcumin and Ustekinumab (CUC) in psoriasis rats exhibits superior efficacy in alleviating psoriatic symptoms and restoring antioxidant levels compared to Ustekinumab alone [8]. Moreover, curcumin demonstrates potent anti-inflammatory properties and has shown effectiveness in treating psoriasis. According to a meta-analysis of 26 studies, CUR, either by itself or in combination therapy, significantly improved patients’ Psoriasis Area and Severity Index (PASI) scores [77]. Preclinical studies also showed that CUR performed better in improving the psoriatic dermatitis phenotype, indicating that it may be a useful treatment for psoriasis [77]. 

Notably, curcumin has been found to elevate levels of crucial antioxidants such as SOD, glutathione peroxidase (GPx), and CAT, highlighting its distinctive antioxidant effects [8]. A study presents a water-responsive gel (WRG) formulation specifically developed to improve the distribution of topical drugs to the skin for the treatment of psoriasis, using curcumin as a model drug. The findings demonstrated that the application of curcumin-loaded WRG (CUR-WRG) showed a notable decrease in oxidative stress indicators, such as MDA, and an increase in antioxidant enzyme activity, including SOD, in both skin tissue and serum. These results emphasize the capacity of CUR-WRG to rectify oxidative imbalance in psoriatic skin, indicating it as a promising method for controlling psoriasis by improving topical antioxidant treatment [78]. An investigation also shows that the administration of curcumin greatly reduced the concentrations of ROS and MDA in skin tissues while simultaneously improving antioxidant defenses, including SOD and CAT [79]. These findings indicate that curcumin has the ability to effectively reduce oxidative damage in psoriasis. 

Research indicates that topical application or oral supplementation of curcumin can effectively alleviate psoriatic symptoms such as scaling, itching, and redness, making it a viable adjuvant treatment option for psoriasis management. A study conducted by Umbert et al. was performed by instructing the patient to apply 2 g of cream (EP2311454) three times a day, spreading it evenly over the afflicted regions and rubbing them in until completely absorbed. Curcumin is one of the antioxidant compounds in EP2311454 therapy, which has been shown to have anti-inflammatory properties in vitro and to be effective in treating patients with mild to severe psoriasis [80]. According to Umbert et al. (2020), clinical outcomes included 80% significant decreases in Dermatology Life Quality Index (DLQI) ratings, a reduction in Body Surface Area (BSA) after 15 days of therapy, and severe patients reaching a PASI of 75. The evidence supports the use of curcumin as a helpful adjuvant therapy for psoriasis symptoms, whether it is used topically or as a supplement. 

In summary, curcumin shows promise as a therapeutic agent for the management of psoriasis due to its strong anti-inflammatory and antioxidant qualities. Research has demonstrated its effectiveness in preclinical models and clinical settings, with improvements observed in BSA, DLQI ratings, and PASI scores. Adding curcumin to therapy regimens, either as an oral supplement or as a topical application, offers a beneficial adjuvant approach to the overall management of psoriasis, potentially relieving symptoms and enhancing the quality of life for patients. Further research is warranted to elucidate its mechanisms of action and optimize its therapeutic use in psoriasis treatment.

### 5.2. Gingerol 

Gingerol, a bioactive compound found in ginger (*Zingiber officinale*), possesses notable antioxidant properties that make it a promising candidate for managing psoriasis symptoms. By neutralizing harmful free radicals in the body, gingerol effectively combats oxidative stress, potentially alleviating the manifestations of psoriasis. Research by Ballester et al. (2022) highlights the pivotal role of oxidative stress in initiating inflammatory pathways such as MAPK, NF-kβ, and JAK-STAT, which subsequently activate immune cells and induce cytokine production, notably TNF-α and IL-17. Ginger’s ability to suppress NF-kβ and reduce cytokine expression underscores its potential as an alternative therapy for psoriasis, particularly given the heightened expression of NF-kβ in psoriatic lesions [81]. The study demonstrates that ginger therapy reduced MDA levels in both erythrocytes and lymphocytes to levels that were even lower than those observed in healthy controls and enhanced the TAS over a period of 60 days, indicating a time-dependent improvement in antioxidant capacity, which may offer therapeutic advantages for controlling oxidative stress [82]. The antioxidant molecule [6]-gingerol, found abundantly in soil ginger, demonstrated antioxidant properties by enhancing CAT activity and decreasing MDA levels in Sprague Dawley rats of various ages [83]. The findings indicate that soil ginger has potential as a nutraceutical for attenuating oxidative stress.

Practically, individuals with psoriasis may find relief through the use of topical therapies containing ginger or by incorporating gingerol-rich dietary supplements or extracts into their regimen. In research by Jenabikordi et al., the encapsulation of metformin and ginger in lipid-based nanoparticles showed promising results in treating psoriasis. Results showed that co-administration of metformin and ginger-loaded liposomes effectively treated psoriatic lesions, significantly reducing inflammatory markers like IL-22 and TNF-α [9]. This study emphasizes the potential of ginger as a supplementary treatment for controlling psoriasis, evidenced by its beneficial impact on the advancement of the illness through the regulation of inflammatory pathways. Moreover, the bioavailability and efficacy of gingerol can be further improved by using novel delivery methods such as nano-encapsulation. These systems safeguard the active chemicals from degradation and allow for precise distribution to the specific tissues that are impacted. Whether applied topically or consumed as a dietary supplement, gingerol offers hope for individuals seeking relief from psoriasis symptoms, although continued research is necessary to fully understand its mechanisms of action.

Despite promising evidence, further investigation is warranted to elucidate the precise mechanisms underlying gingerol’s therapeutic effects in psoriasis. A comprehensive understanding of how gingerol interacts with molecular pathways involved in psoriasis pathogenesis could provide valuable insights into its therapeutic potential and optimize its use in psoriasis management. In conclusion, gingerol, a potent antioxidant compound found in ginger, holds promise as a natural remedy for alleviating the symptoms of psoriasis. Its promise as an alternative treatment for psoriasis management is suggested by its capacity to reduce inflammatory pathways, including NF-kβ, and fight oxidative stress. 

### 5.3. Quercetin (QC)

Quercetin (QC), a natural polyphenol abundant in fruits, vegetables, and herbs, has garnered attention for its potent antioxidant properties and potential therapeutic effects in various diseases, including psoriasis [10]. Its ability to strengthen antioxidant defenses against oxidative stress in psoriasis by scavenging free radicals and upregulating antioxidant enzymes has been well-documented [84]. Given that oxidative stress plays a pivotal role in promoting inflammation and abnormal keratinocyte proliferation, quercetin’s antioxidant properties position it as a promising candidate for managing psoriasis by targeting these underlying mechanisms [85,86]. Studies using an imiquimod-induced psoriasis-like mouse model have demonstrated QC’s potential to offer anti-psoriatic benefits by decreasing PASI scores, alleviating histopathological alterations, and regulating oxidative and inflammatory markers [10]. This study also revealed that quercetin therapy enhances the functions of antioxidant enzymes, including CAT and SOD, while reducing MDA levels [10]. QC has the potential to be used as a natural medicine for the treatment of psoriasis because of its anti-inflammatory and antioxidant qualities [10]. Furthermore, a combination therapy of Cisplatin (cis-dichlorodiammine platinum II) (cDDP) and quercetin has shown promising results, with restored TAS levels and significantly lowered TOS levels indicating QC’s ability to alleviate oxidative stress-related disruptions [87].

Further preclinical research has highlighted the potential benefits of quercetin in treating psoriasis, both topically and through innovative delivery systems. In addition, preclinical research has demonstrated the potential benefit of quercetin in the treatment of psoriasis by improving skin histology and reducing psoriatic symptoms when applied topically [10,85]. For instance, a study aimed to enhance the solubility and anti-psoriatic activity of QC by isolating it from *Psidium guajava* leaves and preparing spanlastics has shown promising results [85]. Spanlastics, known for their biodegradable, biocompatible, and non-immunogenic structure, were utilized as a drug carrier, demonstrating a substantial reduction in Psoriasis Area and Severity Index (PASI) ratings and apoptosis-related gene expression after eight weeks of topical QC spanlastics therapy [85,88]. Using an imiquimod (IMQ)-induced mice model, research has examined the therapeutic potential of QC in psoriasis. The results showcased significant improvements in histology, decreases in PASI scores, and modifications of the NF-κB pathway and pro-inflammatory cytokines [10]. Specifically, QC successfully lowered TNF-α, IL-6, and IL-17 levels, boosted antioxidant enzyme activities, and down-regulated NF-κB pathway components [10]. Clinical trials evaluating formulations containing quercetin, either alone or in combination with other agents, have shown beneficial effects, including reduced erythema, scaling, and plaque thickness.

In summary, with its potent antioxidant and anti-inflammatory properties, quercetin holds promise as a therapeutic agent for treating psoriasis. By targeting oxidative stress and inflammation, quercetin addresses key pathological mechanisms underlying psoriasis pathogenesis. Further research, including well-designed clinical trials, is warranted to fully elucidate the therapeutic potential of quercetin in psoriasis management and to establish its optimal dosage, formulation, and long-term safety profile. However, the evidence suggests quercetin as a possible useful addition to the arsenal of psoriasis therapies, providing a safe and effective natural remedy for those suffering from this crippling skin condition.

### 5.4. Resveratrol (RSV)

Resveratrol (RSV), a natural polyphenol found in red grapes and berries, is recognized for its potent antioxidant properties. Its ability to scavenge free radicals and reduce inflammation positions it as a potential therapeutic agent in diseases like psoriasis, where oxidative stress and inflammation play significant roles [89,90]. Preclinical studies have investigated the efficacy of RSV in psoriasis treatment, yielding promising results that suggest its potential as a novel approach to managing this challenging condition. Preclinical trials exploring RSV’s use in psoriasis treatment have reported reductions in disease severity in mice treated with RSV-based formulations. RSV has demonstrated modulation of various signaling pathways involved in inflammation and immune responses, further contributing to its anti-psoriatic effects. For instance, the development of resveratrol-loaded polymeric micelles (PMs) using a Quality by Design (QbD) approach has shown significant promise in addressing the challenge of poor permeability in psoriatic skin [91]. In an IMQ-induced psoriatic-like plaque model in mice, the optimized PM converted into a gel (PMG) demonstrated superior efficacy compared to conventional gel (CG), with lower levels of hyperkeratosis, serum cytokines, and PASI scores [91]. This suggests that the PMG has potential for improved topical delivery and therapeutic advantages in the treatment of plaque psoriasis. 

RSV has also shown the ability to inhibit the proliferation of keratinocytes, the predominant cell type in psoriatic epidermis. By regulating keratinocyte proliferation, RSV helps restore the balance of cell turnover in the skin, leading to improvements in psoriatic lesions. Studies have demonstrated RSV’s effects on keratinocyte apoptosis and the modulation of signaling pathways involved in psoriasis pathogenesis, along with enhancing antioxidant activity. For instance, RSV treatment raised Sirt1 expression in keratinocytes, leading to inhibition of Akt phosphorylation and apoptosis of human HaCaT cells [92]. A study also exhibits that RSV has antioxidant and anti-proliferative properties on normal human epidermal keratinocytes (NHEKs) by reducing the expression of aquaporin 3 (AQP3), which is excessively produced in hyperplastic skin diseases such as psoriasis [93]. Inhibition of NHEK proliferation by RSV is facilitated by the SIRT1/ARNT/ERK pathway, in which increased SIRT1 expression results in lower ERK phosphorylation and reduced AQP3 expression [93]. The results indicate that RSV has potential as a therapeutic agent for controlling psoriasis by intervening in pathways that control the growth of skin cells.

In addition, a study conducted by Kjær et al. observed the effects of RSV on imiquimod-induced psoriasis-like skin inflammation in mice. RSV modulated IL-17 signaling pathways and mRNA levels of IL-17A and IL-19, suggesting its potential as a treatment for psoriasis [94]. It also significantly decreased the severity of inflammation by decreasing the thickness of the mice’s skin [94]. Despite promising evidence, further research is needed to optimize the dosage, formulation, and long-term safety of RSV in psoriasis treatment. Moreover, extensive clinical trials are warranted to validate its efficacy and assess its potential benefits for different psoriasis subtypes and patient populations. In conclusion, RSV emerges as a natural antioxidant with anti-psoriatic properties, offering potential avenues for further exploration in the management of psoriasis.

### 5.5. Other Antioxidants

Beyond the well-known antioxidant compounds, recent research has identified several other natural compounds that could play a significant role in managing psoriasis by targeting oxidative stress and mtROS. These emerging antioxidants offer new avenues for therapeutic intervention, expanding the potential treatment options for this chronic inflammatory skin condition. For instance, epigallocatechin-3-gallate (EGCG), the flavonoid compound from green tea, has also demonstrated the ability to suppress mtROS production and enhance mitochondrial function by upregulating antioxidant defenses and reducing pro-oxidant enzyme activity [95]. These compounds collectively target oxidative stress and mtROS, offering promising avenues for improving psoriatic outcomes through natural antioxidant therapy. EGCG exhibited its antioxidant properties in psoriasis by reducing MDA concentrations, which serve as an indicator of oxidative stress, and enhancing the effectiveness of antioxidant enzymes such as SOD and CAT [96]. This study also discovered that applying EGCG topically improved skin structure, decreased T-cell infiltration, and reduced pro-inflammatory cytokines such as IL-17 and IL-23, thereby alleviating psoriasis-like symptoms in imiquimod-induced mice [96]. Other than that, a study also shows that EGCG effectively decreases MDA levels in HaCaT cells that are subjected to UV light. The optimized formulation of EGCG with hyaluronic acid exhibited the most significant decrease in MDA levels, suggesting a more potent inhibition of UV-induced oxidative damage in comparison to plain EGCG and EGCG-transfersomes [97]. The results emphasize the prospective of EGCG as a potent natural antioxidant in controlling oxidative stress and inflammation in psoriasis.

In addition, rutin is a hydrophobic polyphenolic constituent of the flavonoid family found in several foods, including citrus, apples, buckwheat, black tea, and green tea [98]. Numerous studies demonstrate that the inherent antioxidant characteristics of rutin may contribute to the mitigation of oxidative stress and inflammation, pivotal elements in the pathogenesis and advancement of psoriasis. A study provides evidence that rutin effectively mitigates psoriasis-like symptoms in a mouse model generated by imiquimod by decreasing inflammation, severity of skin lesions, and oxidative stress. The utilization of a melatonin and rutin ointment topically successfully reduced the concentrations of inflammatory markers such as TNF-α and IL-17A and decreased MDA, suggesting its potential as a therapeutic intervention for psoriasis [99]. The results were similar to those of clobetasol, a conventional therapy, indicating that rutin shows promise as a natural alternative for the management of psoriasis. A study also demonstrates that rutin efficiently safeguards keratinocytes against oxidative stress by decreasing the concentrations of ROS, nitric oxide (NO), and MDA while simultaneously increasing the activity of SOD and GSH-Px [100]. Furthermore, rutin reduced the synthesis of inflammatory cytokines (IL-6, IL-1β, IL-23A) and stimulated the Nrf2 pathway, therefore enhancing the expression of antioxidant enzymes NQO1 and HO-1 [100]. Additionally, a study demonstrates that the combination of ascorbic acid and rutin provides enhanced antioxidant protection against UV-induced skin damage in keratinocytes and fibroblasts. The combined treatment significantly increased the activity of antioxidant enzymes, including CAT and SOD [101]. The concurrent therapy successfully decreased the production of ROS, reduced the oxidation of lipids and proteins, and targeted pro-inflammatory and pro-apoptotic pathways, including NFκB and caspases [101]. These findings indicate that the combination of ascorbic acid and rutin has strong cytoprotective, anti-inflammatory, and anti-apoptotic properties, making them a promising method for to minimize oxidative stress in psoriasis. All of these results indicate that rutin has the potential to alleviate oxidative stress associated with psoriasis by strengthening the skin’s antioxidant mechanisms.

Genistein is a flavonoid commonly present in plant-based sources, including soybeans. Several studies have extensively demonstrated a wide range of biological effects, including antioxidant and anticancer activities [102]. The anti-psoriatic properties of genistein are attributed to its capacity to decrease the proliferation of keratinocytes, inhibit the nuclear translocation of NF-κB, and regulate inflammatory reactions. The results of a study indicate that genistein may enhance abnormal gene expression associated with psoriasis and decrease cytokine synthesis, thereby justifying additional investigation into its possible therapeutic application [103]. Genistein may serve as an effective treatment for psoriasis by blocking cytokine-induced NF-κB activation and reducing key inflammatory cytokines such as IL-8, IL-20 and CCL2 [103]. In addition, a study also noticed in vivo studies that genistein, at dosages of 50 and 100 M for 2 h in a mouse model of imiquimod-induced psoriasis, leads to a decrease in the levels of cytokines, including IL-1, IL-6, TNF-α, CCL2, IL-17, and IL-23 [104]. Genistein also demonstrates promise in the treatment of psoriasis by decreasing the severity of skin lesions and the thickness of the epidermis in an imiquimod-induced animal model [104]. Furthermore, it hinders the growth of keratinocytes and reduces the activity of the NF-κB and STAT3 signaling pathways, indicating its potential therapeutic function in the management of psoriasis [104]. Furthermore, Wang et al. (2010) conducted a prior investigation which showed that genistein therapy increased the activity of SOD and decreased the levels of MDA, suggesting a transition towards a more favorable oxidative equilibrium in the cells [105]. The promise of genistein as a therapeutic agent for psoriasis is demonstrated by its ability to reduce oxidative stress, lower levels of inflammatory cytokines, decrease the proliferation of keratinocytes, and modulate important signaling pathways. Therefore, further research is necessary to explore its application in the management of the condition.

## 6. Discussion and Prospects

The role of mtROS in psoriasis has emerged as a key area for understanding the disease’s pathophysiology. Disruption of mtROS perturbs cellular equilibrium, leading to oxidative stress, DNA damage, and mitochondrial dysfunction, all of which are crucial for the development and advancement of psoriasis [8,9,10]. These mechanisms not only induce oxidative stress but also stimulate inflammatory reactions, hence sustaining the cycle of the disease. A comprehensive grasp of these molecular processes is crucial for the development of therapeutic approaches that specifically address oxidative stress and inflammatory pathways. 

The characterization and utilization of oxidative stress indicators, including MPO [22,23,25,26,27,28,29,70], PON [23,30,31,32,34,35,36,37,38,39,40], SIRT [41,42,43,44,45,46,47,48,49,50,51], SOD [38,52,53,54,55,56,57,58], and CAT [38,59,60,61,62,63,64,65] provide novel understanding of the development of psoriasis. These biological markers aid in evaluating the levels of oxidative stress and inflammation, thereby functioning as possible diagnostic instruments and indications for the severity and advancement of diseases. Furthermore, they have the capability to forecast individual reactions to therapies, therefore facilitating the implementation of more tailored therapeutic strategies. Further investigation and verification of these indicators have the potential to completely transform the diagnosis and treatment of psoriasis by providing precise guidance for medicines that focus on and address specific oxidative stress pathways.

Furthermore, it is essential to comprehend oxidative stress markers such as MDA [50,56,66,67,68,69,71], OSI [56,72,73,74], TOS [4,8,11,56,74], and TAS [2,8,11,74,75,76] in order to assess the overall redox condition in individuals with psoriasis. Elevated levels of MDA, which indicate lipid peroxidation and oxidative damage, are linked to the severity and advancement of the disease. Elevated levels of TOS indicate heightened oxidative stress, whilst reduced levels of TAS imply a compromised antioxidant defence network. Analysis of the OSI, which quantifies the equilibrium between TOS and TAS, is associated with disease activity, rendering it a useful indicator for tracking therapy response and informing therapeutic choices. The implementation of treatments that focus on restoring redox equilibrium and decreasing oxidative stress has the potential to greatly enhance therapeutic results in individuals with psoriasis.

Future research should prioritize the clarification of the processes by which mtROS contribute to the development of psoriasis and their connections with mitochondrial dysfunction, oxidative stress, and immunological responses. Potent natural antioxidants, including curcumin [8,77,78,80], gingerol [9,81,82,83], quercetin [84,85,86], resveratrol [89,90,91,92,93,94], epigallocatechin-3-gallate (EGCG) [95,96,97], rutin [98,99,100,101], and genistein [102,103,104,105], have demonstrated potential in mitigating oxidative stress and inflammation, while simultaneously strengthening antioxidant defences. These compounds have demonstrated efficacy in preclinical studies by lowering oxidative stress markers, boosting antioxidant enzyme activities, and alleviating psoriatic symptoms. Future research should refine these therapies by exploring optimal dosages, formulations, and delivery methods to maximize therapeutic benefits while minimizing side effects.

In addition, clinical trials play a vital role in confirming the safety and efficacy of natural antioxidants as independent pharmaceuticals or when used with current treatments. Advancements in drug delivery technologies, such as nano-encapsulation and gel formulations, have the potential to significantly improve their bioavailability and treatment efficacy of these compounds, providing more targeted and effective therapies [9,78]. A more holistic strategy to managing psoriasis and enhancing patient outcomes could be achieved by investigating the synergistic effects of combining conventional medications with antioxidant therapy. Incorporating natural antioxidants into psoriasis therapy modalities, in conjunction with progress in biomarker detection and a more profound comprehension of oxidative stress, has significant potential for improving patient care. Further investigation in this area has the potential to result in more efficient, secure, and tailored treatments, thereby offering optimism for enhanced patient care and an elevated standard of living for individuals impacted by this complex dermatological condition.

## 7. Conclusions

In conclusion, the intricate interplay between ROS production, mitochondrial dysfunction, and the pathophysiology of psoriasis underscores the significance of targeting mitochondrial ROS as a potential therapeutic strategy. Harnessing antioxidants and natural products to counteract mitochondrial dysfunction holds promise for revolutionizing psoriasis treatment. Integrating these alternative therapies into current treatment protocols could unveil novel avenues for symptom management and enhance patient outcomes. However, comprehensive investigation of mitochondrial ROS biology in psoriasis and rigorous validation of the efficacy and safety profiles of natural products are essential. These initiatives will open the door to individualized care catered to the special requirements of psoriasis sufferers, thereby enhancing treatment results and quality of life.

## Data Availability

Not applicable.
